# The Missing Link: A Case of Plummer-Vinson Syndrome in a Young Pacific-Islander Woman With Helicobacter Pylori

**DOI:** 10.7759/cureus.18934

**Published:** 2021-10-20

**Authors:** Adham E Obeidat, Shirley So, Joseph Go, Traci T Murakami

**Affiliations:** 1 Internal Medicine, University of Hawaii, Honolulu, USA; 2 Internal Medicine, California Pacific Medical Center, San Francisco, USA; 3 Department of Gastroenterology and Hepatology, The Queen's Medical Center West Oahu, Ewa Beach, USA

**Keywords:** helicobacter pylori, pacific-islander, upper endoscopy, esophageal web, iron deficiency anemia (ida), dysphagia

## Abstract

Plummer-Vinson syndrome (PVS), the triad of dysphagia, iron-deficiency anemia (IDA), and esophageal webs, is a relatively rare disease entity that is mostly observed in the Caucasian populations of Scandinavia and North America. As these regions have become more developed with improved nutrition, PVS is now more commonly seen in the developing regions of the world.

We present the case of a 29-year-old Pacific-islander woman who presented with progressive dysphagia and IDA and was found to have an esophageal web and *Helicobacter pylori* (*H. pylori*) gastritis on upper endoscopy. She improved with dilation of the web in the esophagus and treatment of *H. pylori*. Identifying the possibility of this syndrome in clinical practice and the association between *H. pylori *and PVS, especially given recent changes in its epidemiology, is important given the patient population in Hawaii and the Pacific.

## Introduction

Plummer-Vinson syndrome (PVS) is a rare syndrome characterized by the triad of dysphagia, iron-deficiency anemia (IDA), and proximal esophageal webs, which are thin membranes that may develop across the lumen of the esophagus [1-3]. It was first reported in 1912 by Henry Stanley Plummer, who described it as upper esophageal spasms without anatomic stenosis. In 1919, Porter Paisley Vinson described dysphagia due to an “angulation” of the esophagus and treated it with esophageal dilation [4,5]. The disease is also known in the United Kingdom as the Paterson-Brown-Kelly syndrome, named after British laryngologists Donald Ross Paterson and Adam Brown-Kelly, who published the signs and symptoms of the syndrome independently in 1919 [6,7].

Modern-day epidemiological data about the incidence and prevalence of PVS is not widely available. A single population-based study in the 1960s in South Wales reported the prevalence of post-cricoid webs was between 8.4% and 22.4% in women with dysphagia, while no webs were identified in men [8]. In recent years, there have also been more cases of men with PVS [9,10].

PVS is most common in middle-aged women, which accounts for almost 90% of cases [1-3]. The high prevalence in women is thought to be due to inadequate dietary intake and chronic blood loss from menstruation and pregnancy, causing IDA [1,3]. Initially, PVS cases were reported mainly in Europe and North America, especially in Scandinavia. However, given economic growth, improvement in nutrition and hygiene, reduced parasitic infestation, and iron supplementation in these regions, the condition has almost disappeared. On the other hand, PVS has been increasingly recognized in the developing countries of Asia. However, there is a lack of reported cases of PVS among Pacific islanders [9,11,12]. 

The initial management of PVS involves iron and B-complex supplementation. In most cases, iron therapy alone results in the improvement of symptoms [3]. Moreover, endoscopic dilation can be performed in patients with persistent dysphagia [1,2]. PVS can be associated with an increased risk for upper gastrointestinal (GI) tract cancers, especially squamous cell carcinoma of the hypopharynx and proximal esophagus; therefore, regular surveillance with esophagogastroduodenoscopy (EGD) is recommended [1,3,11,12]. We present the case of a 29-year-old Pacific-islander woman who presented with progressive dysphagia and IDA and was found to have an esophageal web and *Helicobacter pylori* (*H. pylori*) gastritis on EGD.

## Case presentation

A 29-year-old Pacific-islander (Chuukese) woman with no past medical history presented to the GI clinic for evaluation of dysphagia. She started having progressive difficulty swallowing nine months before the evaluation, mainly for solid food. She lost 78 pounds in three years. She also reported heartburn, intermittent chest and throat pain, and nausea but denied vomiting, abdominal pain, diarrhea, constipation, or bloody stool. She visited the emergency room one week prior for fatigue and was diagnosed with microcytic anemia.

**Figure 1:**
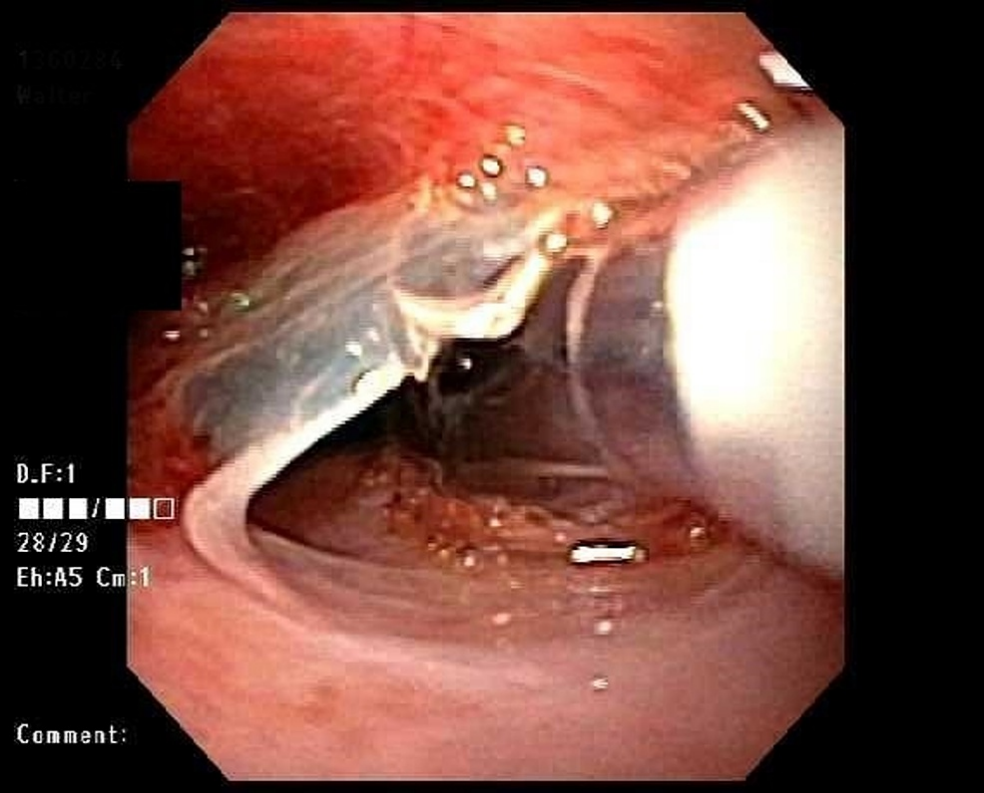
Endoscopic image demonstrates a proximal esophageal web.

In the GI clinic, physical examination was unremarkable, including an abdominal exam. Initial laboratory studies were significant for microcytic anemia with a hemoglobin of 11.1 g/dL and a mean corpuscular volume (MCV) of 78.6 FL. Otherwise, the white blood cell count (WBC), platelet count, kidney function test, and liver function test were normal. Iron studies were consistent with IDA with a total iron level of 25 ug/dL, low saturation, high iron-binding capacity, and ferritin level of 8 ng/mL. A barium esophagogram was performed and revealed no abnormalities. Therefore, the patient underwent an EGD, which revealed a stricture from an esophageal web in the proximal esophagus (Figure 1), which was dilated. Biopsies of the middle and lower thirds of the esophagus, the stomach, and the duodenum were taken. Pathology revealed normal esophageal mucosa with chronic inflammatory changes, *H. pylori* chronic gastritis, and increased intraepithelial lymphocytes in the duodenum.

The patient was started on triple therapy (amoxicillin, erythromycin, and proton pump inhibitor) for treatment of *H. pylori* infection as well as supplementation with ferrous sulfate for her IDA. She was scheduled for another EGD a month later. The results of the EGD showed improved esophageal stenosis caused by the proximal esophageal web, which was dilated again, and biopsies were taken from the proximal esophageal web. Pathology showed normal squamous mucosa with no evidence of histopathologic abnormalities. The patient was scheduled for a follow-up EGD, but she was lost to follow-up.

## Discussion

PVS has been reported from all parts of the world but was initially concentrated in Northern European countries [1,2,13]. Currently, PVS is increasingly recognized in developing countries of Asia, such as India [9]. However, there is still a lack of reported cases of PVS among Pacific islanders. Most PVS patients are Caucasian, middle-aged women aged 40- to 70-years-old. Notably, there is an extremely high prevalence of IDA in children aged 1-5 years living in American Samoa [13].

Dysphagia is the most common presenting symptom of PVS, resulting from luminal stenosis and esophageal dysmotility. It is often low-grade, intermittent, and painless. The progression of dysphagia is slow and can take several years [12]. Patients also often have IDA, which can present with non-specific signs and symptoms. Iron deficiency is more important than the presence of anemia and is not necessarily hypochromic and microcytic [14]. Esophageal webs are the characteristic finding in PVS and usually occur in the proximal esophagus at the post-cricoid level [1,2]. Our patient presented with dysphagia along with IDA and was found to have proximal esophageal webs on EGD, which is characteristic of PVS.

Although the pathogenesis of PVS is unclear, there are several proposed mechanisms, including nutritional deficiency (iron, vitamin B), genetic predisposition, and an autoimmune process [1,3]. It is purported that IDA causes the reduced activity of iron-dependent oxidative enzymes resulting in mucosal degeneration and myasthenic changes, leading to damages in the esophageal mucosal epithelia and subsequent dysphagia [1-3,15]. However, this fails to explain the occurrence of esophageal webs in patients without IDA, lack of global response to iron supplementation, and the low cases of PVS in Africa, despite a high prevalence of IDA [1,2]. Other nutritional deficiencies, such as riboflavin, thiamine and pyridoxine deficiency, have also been thought to contribute. Autoimmunity is thought to play a role, as PVS is associated with other autoimmune conditions, such as celiac disease, rheumatoid arthritis, and Crohn’s disease. Furthermore, chronic inflammation from a variety of factors has been proposed as part of the pathogenesis [1,2,11,12]. *H. pylori* infection impairs iron absorption as a result of chronic gastritis, which causes gastric hypochlorhydria and subsequent impaired iron uptake and an increase in iron loss, potentially leading to IDA. One case report from Japan described successful therapy of PVS with endoscopic dilation and eradication of *H. pylori *infection [16].

Barium esophagram is the most sensitive diagnostic test to detect esophageal webs. However, it can be unrevealing, and therefore endoscopy is required to make the diagnosis. Endoscopy should be done cautiously as the web is usually detected close to the upper esophageal sphincter and thus can be missed or ruptured during the passage of the endoscope [1,2]. 

The first step in the management of PVS is to clarify the etiology of the IDA by excluding other causes, such as celiac sprue, malignancy, or hemorrhage. The initial management of PVS involves iron and B-complex supplementation [3,15]. In one retrospective study, eight out of 50 cases presenting with IDA, dysphagia, and esophageal webs were treated successfully with iron supplementation alone with a complete resolution of symptoms [17]. However, long-standing dysphagia is unlikely to respond and will need esophageal dilation [1,2]. Dilation can be performed using balloon dilation or Savary-Gilliard dilators. Endoscopic laser division and electro incision have also been used, and surgery is only needed in rare refractory cases [1,18,19]. In a prospective study of 30 patients with PVS, a single session of dilation provided a complete response in 90% of patients [2]. Recurrence of dysphagia is low and even lower with balloon dilation compared with Savary-Gilliard dilation [1,9]. Our patient’s dysphagia improved slightly with iron supplementation, and she underwent balloon dilation with subsequent improvement, but unfortunately, she was lost to follow-up.

PVS has an excellent outcome; however, it can be associated with an increased risk for upper GI tract cancers. There have also been reports of gastric cancer. Thus, regular follow-up with endoscopic surveillance for this premalignant condition is recommended even after the resolution of symptoms [1,3,11,12]. 

## Conclusions

PVS is a rare finding among Pacific islanders. Our patient was a young Chuukese woman, which is not the usual demographic of PVS patients. Our case provides a fresh new perspective on the etiology of PVS and its possible link to *H. pylori* infection, and thus evaluation of patients with PVS should also include testing for and treatment of *H. pylori*. Further studies are needed to clarify the association between *H. pylori* and PVS, and perhaps this may be the origin of iron deficiency in PVS and the association between PVS and gastric malignancy. The diagnosis of PVS requires a high clinical suspicion. Thus, for any patient with dysphagia and IDA, PVS should be on the differential diagnosis as it carries an increased risk of upper GI malignancies and responds well to treatment with iron supplementation and esophageal dilation.
